# Natural high ambient temperature-induced respiratory hypocapnia without activation of the hypothalamic-pituitary-adrenal axis in lactating goats

**DOI:** 10.14202/vetworld.2022.2611-2616

**Published:** 2022-11-19

**Authors:** Sapon Semsirmboon, Dang Khoa Do Nguyen, Narongsak Chaiyabutr, Sutthasinee Poonyachoti, Sumpun Thammacharoen

**Affiliations:** 1 Department of Physiology, Faculty of Veterinary Science, Chulalongkorn University, Pathumwan, Bangkok 10330, Thailand; 2 The Academy of Science, The Royal Society of Thailand, Dusit, Bangkok 10300, Thailand; 3 Queen Saovabha Memorial Institute, The Thai Red Cross Society, Bangkok 10330, Thailand

**Keywords:** acid-base imbalance, lactation, Saanen goat, thermal panting, tropical climate

## Abstract

**Background and Aim:**

Activation of breathing, the hypothalamic-pituitary-adrenal (HPA) axis, and plasma antioxidant defense are adaptive mechanisms in lactating dairy goats fed during the summer season. However, an excess of these responses can interfere with the gas exchange. This study aimed to investigate the effect of natural high ambient temperature (HTa) on blood gas parameters and their relation to the HPA axis and antioxidant defense.

**Materials and Methods:**

Six mid-lactating goats were included in this study and were fed in individual pens for 2 weeks. The data on ambient conditions, physiological responses, and blood chemistry were measured for two sampling days (D7 and D14), 1 week apart during the late summer season. On this two-sampling day, the main physiological responses to HTa, including respiration rate (RR), rectal temperature (Tr), blood gas, and blood chemistry, were measured in the morning and afternoon.

**Results:**

Goats from both D7 and D14 increased RR and Tr significantly according to morning and afternoon periods. In addition, goats were at the hypocapnia stage during afternoon panting without a change in blood pH and bicarbonate levels. Interestingly, HTa-induced hypocapnia was not accompanied by an increase in plasma cortisol levels. Finally, ΔTa was negatively correlated with changes in glutathione peroxidase activity.

**Conclusion:**

The natural HTa (ΔTa; 5−8°C) in this study activated evaporative heat dissipation and was high enough to induce respiratory hypocapnia. Importantly, this ΔTa did not activate the HPA axis but was correlated with a change in antioxidant defense. Therefore, under natural HTa in tropical conditions, respiratory hypocapnia is the first line of physiological response in goats within a specific range of natural ΔTa (5−8°C).

## Introduction

Tropical savannah is the predominant climatic condition in Thailand [1]. The year-round high ambient temperature (HTa) is the main characteristic of this climate type. The adaptive mechanism of dairy goats to HTa was in part mediated through the activation of panting, the hypothalamic-pituitary-adrenal (HPA) axis, and plasma antioxidant defense [2–4]. Under the HTa of the climatic chamber, an excess of these responses interfered with the blood gas parameters in goats [3, 5, 6]. These indicate that the natural HTa could negatively affect lactating goats.

It is known that HTa negatively affects the milk production of dairy goats. During the daytime in Thailand HTa, dairy goats showed increases in both rectal temperature (Tr) and respiration rate (RR) [2, 4, 7]. These responses indicate the activation of evaporative heat dissipation. In addition, plasma cortisol levels appeared to increase during afternoon HTa. This suggested that lactating dairy goats fed HTa were under heat stress [2, 4]. In the early phase of the response, dry matter intake (DMI) was decreased to limit heat production [2, 5, 6]. Likewise, water intake (WI) was increased to maintain body water and evaporative heat dissipation [5, 8]. In addition, the response to HTa resulted in changes in blood chemistry and gas parameters. Changes in blood chemistry, including pack cell volume (PCV) and glucose, have been consistently reported in ruminants fed HTa in both natural and climatic chambers [3, 5, 8]. When goats were exposed to a high degree of HTa with the difference between control Ta and HTa (ΔTa) at 15°C, excess panting appeared with altered blood gas parameters, including blood pH, partial pressure carbon dioxide (PCO_2_), and bicarbonate (HCO_3_) [3, 5, 6]. These responses were observed with changes in plasma cortisol and antioxidant levels in a climatic chamber [3]. However, the natural short-term HTa during daytime in tropical conditions is approximately 10°C [2, 7]. Even though this ΔTa (10°C) was lower than climatic conditions, it was enough to increase evaporative heat dissipation by panting in the lactating goat.

The low degree of HTa in natural climate inspired us to investigate whether the natural HTa could influence blood gas homeostasis and whether this response was related to either the HPA axis or antioxidant defense. Therefore, this study aimed to investigate the effect of short-term natural HTa on blood gas parameters, the HPA axis, and antioxidant defense of lactating goats fed under tropical conditions.

## Materials and Methods

### Ethical approval

All procedures followed the guidelines of the Ethical Principles and Guidelines for the Use of Animals for Scientific Purpose of the National Research Council of Thailand and were approved by the Institutional Animal Care and Use Committee (IACUC) in accordance with the University regulations and policies governing the care and use of experimental animals (animal use protocol no. IACUC 1931102).

### Study period and location

The study was conducted from June to July 2021. The study was conducted on commercial dairy goats in Nan Province, Thailand (latitude 18.81772°, longitude 100.77350°).

### Animals and meteorological data

Six mid-lactating Saanen goats aged 3 years were included in this study. The average body weight and milk yield were 43 ± 2.3 and 1.7 ± 0.2 kg, respectively. The goats were fed and observed in individual pens (90 × 200 cm) in the late summer season for 2 weeks. The data and samples were collected at the end of each week (D7 and D14) to cover the variable ΔTa in the early morning and afternoon of the summer. All goats were fed a concentrate diet (Mamae Lactating goat feed, Lopburi, Thailand) based on milk yield and divided into two meals scheduled at 0600 and 1300. Roughage and water were provided *ad libitum*.

The ambient temperature (Ta, °C) and humidity (RH, %) in the goat barn were measured using a wet–dry bulb. These data were recorded thrice daily, at 0800, 1200, and 1800. Moreover, data from the last two points were averaged to represent afternoon Ta, while ΔTa was calculated by subtracting morning Ta from afternoon Ta. The temperature and humidity index (THI) was calculated according to the National Research Council [9]. The equation used is as follows:

THI = ((Tdb + Twb) * 0.72) + 40.6

Where; Tdb = dry bulb temperature and Twb = wet bulb temperature.

### Data collection, measurement, and analysis

Concentrate and roughage were sampled daily and kept at −20°C for later chemical analysis. All samples were pooled and analyzed according to AOAC 1990 [10]. The chemical composition of the feed is presented in Table-1. To study HTa responses, the parameters including Tr, RR, DMI, WI, and urine volume (UV) were also measured on two sampling days (D7 and D14), and each measurement was detailed. Rectal temperature was measured by placing a thermometer against the rectum, whereas RR was measured by counting the movement of the chest within 1 min. Both parameters were measured at 0800, 1200, and 1800. In addition, the DMI and WI were measured by subtracting refusal from the offer. Finally, UV was measured using a weight urine bucket placed beneath each pen.

**Table-1:** Chemical compositions of the feed component (% on dry matter basis).

Chemical compositions (%)	Hay	Concentrate
Moisture	7.32	8.74
Dry matter	92.68	91.26
Crude protein	3.87	14.67
Fat	0.95	3.57
Ash	7.15	6.50
Neutral detergent fiber	73.12	-
Acid detergent fiber	44.84	-

Blood samples (5 mL) were collected from the jugular vein at two sampling times, 08:00 and 15:00, on both sampling days. Each sample was then separated into two parts. The first part was used to measure pH, PCO_2_, HCO_3_, glucose, and PCV with an iSTAT blood gas analyzer (Abbott, New Jersey, USA) within 30 min of collection. To obtain this part, blood was drawn using a 1 mL heparinized syringe with a cap and placed on crushed ice until analysis. The second part contained 4 mL of blood stored in a lithium-heparin blood tube. To harvest plasma, blood was centrifuged at 12000 g at 4°C for 10 min, then kept under −80°C for cortisol, glutathione peroxidase (GPx) activity, and malondialdehyde (MDA) measurement. The plasma cortisol levels were determined using a competitive enzyme immunoassay technique (CBS-E18048G, CUSABIO, Houston, USA). The plasma cortisol competed with pre-coated antigen, then the strong reaction indicated a lower amount of plasma cortisol. In addition, both GPx activity and MDA concentration were measured by colorimetric method using commercial kits. These kits were GPx assay kit (ab102530, Abcam, Cambridge, USA) and lipid peroxidation (MDA) assay kit (ab118970, Abcam, Cambridge, USA). To measure GPx activity, plasma was mixed with the colored GPx substrate, and the reduction rate of the substrate represented this enzyme activity. In contrast, the high plasma MDA formed a high level of colored product (MDA-Thiobarbituric acid adduct) in the reaction buffer of the commercial kits.

### Statistical analysis

All data are presented as mean ± standard error of the mean. The normality of data was screening using Shapiro–Wilk test and all data met the assumption. The difference in one factor that contained two data sets was tested using Student’s t-test. Data containing two factors were analyzed using repeated two-way analysis of variance, and the Bonferroni test was used for *post hoc* analysis. Statistical significance was declared at p < 0.05.

## Results

### Ambient condition and the effect of natural HTa on Tr, RR, DMI, WI, and UV

The Ta of this current increased during the day, and the peak Ta was found at 1300. The morning Ta of two-sampling day was 25°C, while the afternoon Ta was 33°C and 30°C on D7 and D14. Then, the ΔTa in early morning and afternoon (daytime HTa) of both sampling days was 8 and 5°C. In addition, the ranges of daytime RH on this two-sampling day were 56−77% and 76−90%, respectively. According to Ta and RH, the calculated daytime THI ranged from 75−87 and 76−82 on D7 and D14, respectively.

**Figure-1:**
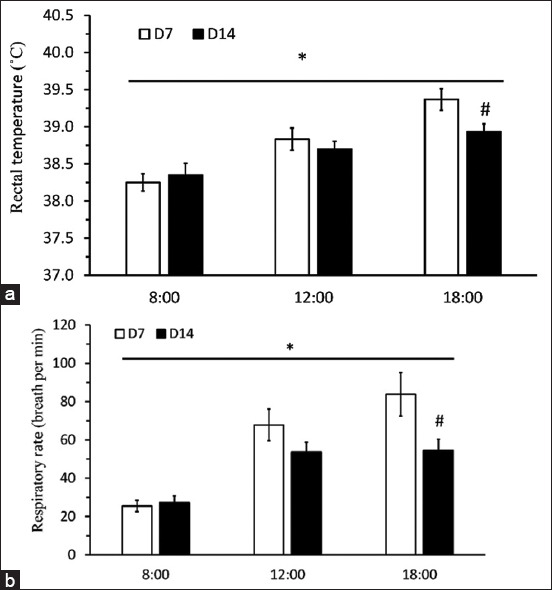
(a) The average rectal temperature (°C) and (b) the average respiration rate (breath per min) from day 7 (open bar) and day 14 (dark bar) at 8:00, 12:00, and 18:00. *The significant difference by two-way repeated analysis of variance (p < 0.05) and ^#^the significant difference determined using Student’s t-test (p < 0.05).

The effect of sampling day was not found on either Tr or RR, whereas daytime HTa significantly affected both parameters (Figures-1a and b; F_2,20_ = 65.02, F_2,20_ = 29.49, p < 0.05). Average Tr in the afternoon was significantly higher than that in the early morning. In addition, a difference in Tr between D7 and D14 was found at 1800 (T_5_ = 3.135, p < 0.05). In addition, there was no effect of sampling day on concentrate intake, roughage intake, WI, and UV (Table-2; T_5_ = 1.133, 0.4248, 1.170, and 2.002, p > 0.05).

**Table-2:** The effect of natural HTa on concentration intake, roughage intake, water intake, and urine volume from D7 and D14.

Items	D7	D14	p-value
Concentrate intake (gDM/kgBW/day)	19 ± 0.7	19.2 ± 0.7	0.31
Roughage intake (gDM/kgBW/day)	12 ± 0.8	11.5 ± 1.4	0.69
Water intake (mL/kgBW/day)	112.6 ± 9.2	104.2 ± 12.4	0.15
Urine volume (mL/kgBW/day)	24.8 ± 6.1	32.0 ± 7.2	0.10

HTa=High ambient temperature, D7=Day 7, D14=Day 14

### The effect of natural HTa on blood gas parameters, blood chemistry, cortisol, and antioxidant capacity

**Figure-2:**
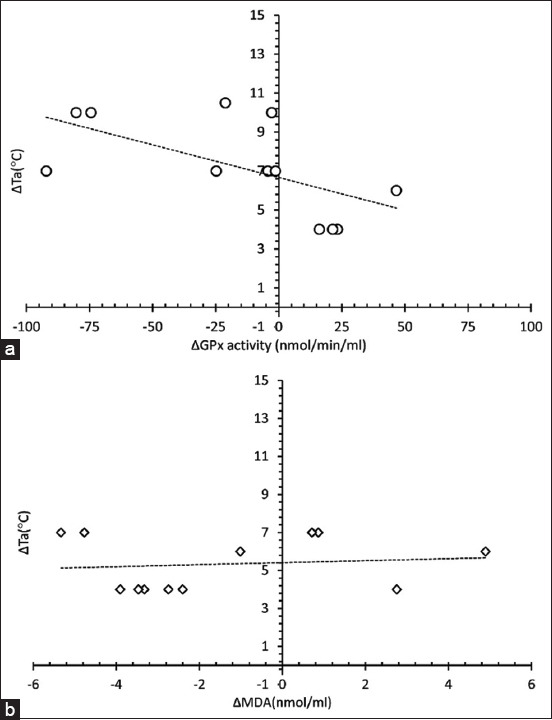
(a) The correlation of between a change of plasma glutathione peroxidase activity (ΔGpx) and (b) malondialdehyde (ΔMDA) with ΔTa. The significant correlation was determined using Pearson correlation (p < 0.05).

There was no effect of sampling time on blood pH, PCO_2_, and HCO_3_ (Table-3; F_1,10_ = 0.01, 0.001, and 0.01, p > 0.05), whereas the effect of daytime HTa was found only on PCO_2_. The average PCO_2_ from two sampling days in the morning and afternoon was 40.5 ± 0.5 and 39.2 ± 0.6 mmHg, respectively. In addition, no significant effect of the sampling day was found on plasma glucose, PCV, and cortisol (Table-3). However, there was a significant effect of daytime HTa on blood glucose (Table-3; F_1,10_ = 38.44, p < 0.05), and the average blood glucose levels from the two-sampling days in the morning and afternoon were 55 ± 1.1 and 60 ± 0.8 mg/dl. Furthermore, there was a negative correlation between ΔTa and a change in GPx activity (Figure-2a; r = 0.6, p < 0.05), whereas ΔTa was not correlated with a change in plasma MDA concentration (Figure-2b; r = 0.3, p > 0.05).

**Table-3:** The effect of natural HTa on blood parameters from D7 and D14.

Items	D7		D14		SEM	p-value		
		
	8:00	15:00	8:00	15:00		Day	Time	Day X time
Blood pH	7.44	7.44	7.42	7.46	0.02	0.93	0.05	0.02
Blood HCO_3_ (mmol/l)	27.58	26.45	26.57	27.70	1.15	0.97	0.02	0.85
Blood PCO_2_ (mmHg)	40.47	39.18	40.58	39.12	1.44	0.94	0.99	0.08
Blood glucose (mmol/l)	3.13	3.29	2.97	3.38	0.11	0.68	0.001	0.03
PCV (%)	21	21.2	20.7	19.8	0.87	0.56	0.34	0.17
Cortisol (nmol/l)	59.5	67.4	78.1	61.7	20.2	0.75	0.65	0.22

SEM=Standard error of the mean, HTa=High ambient temperature, D7=Day 7, D14=Day 14

## Discussion

This study demonstrated the effects of natural HTa on blood gas parameters in dairy goats fed under tropical conditions. The investigation focused on the HTa condition in the afternoon compared with the control Ta in the early morning. The natural HTa with ΔTa at 5−8°C increased Tr and RR, which indicated the activation of evaporative heat dissipation. These responses induced respiratory hypocapnia without changes in blood pH, HCO_3,_ and HPA axis. Interestingly, the ΔTa in the morning and afternoon in this investigation was related to a change in antioxidant defense.

According to meteorological data, dairy goats in the current investigation were fed in hot and humid climates. The ΔTa at 5−10°C during the daytime was in line with other previous publications [2, 4]. This natural HTa consistently increased the Tr and RR of dairy goats compared to those in the early morning [4]. The difference of ΔTa between summer and winter was 3°C; this difference was enough to demonstrate the effect of HTa on Tr, RR, and DMI [2]. In addition, plasma cortisol was increased in the afternoon, and this response indicated the activation of the HPA axis [4]. In this study, the difference of ΔTa between two sampling days at 3°C (ΔTa = 5 and 8°C) did affect Tr and RR. However, this difference did not affect the DMI and plasma cortisol levels. The data indicate that the different degrees of heat dissipation between the two sampling days and the activation of the HPA axis were not responsible for this response. Heat dissipation requires water that is consumed by drinking water and water in the feed [11]. Interestingly, the different degrees of heat dissipation were not accompanied by changes in the WI or DMI in this study. Moreover, neither UV nor PCV were affected by daytime HTa. These observations were compared to the early phase of the HTa response, which is not involved in the stress response [4]. In rats, the early HTa response is involved in the shift of water from the extravascular compartment to blood vessels. Therefore, this mechanism may be responsible for the different degrees of heat dissipation at this natural HTa (ΔTa = 5−8°C).

Panting is the major physiological response to evaporative heat dissipation in lactating dairy goats fed under tropical conditions [2]. Excess panting affects acid-base balance and results in respiratory hypocapnia [3, 12]. At this stage, CO_2_ exhalation increased while PCO_2_ decreased. When humans were exposed to a mild degree of hypocapnia, blood pH and HCO_3_ were not affected. If the degree of hypocapnia is further increased, the blood pH and HCO_3_ levels eventually change [13]. Blood PCO_2_ and HCO_3_ are the major acids and bases in the blood. The loss of acid by respiratory hypocapnia is compensated by decreased HCO_3_ in the blood [13]. In humans, this compensation can be classified into two stages: acute and chronic [13]. In an acute response, the intracellular buffer system is responsible for this compensation [14]. In addition, reabsorption of HCO_3_ by the kidney is mainly responsible for the chronic response to respiratory hypocapnia [15]. These responses were also demonstrated in goats fed under HTa in a climatic chamber [3, 5], where ΔTa was 15°C. This climatic condition did increase Tr by 1−2°C and increase RR by 3−5 times compared to the control temperature. However, the natural HTa of tropical savannah increased Tr by 0.5–1°C and doubled RR when compared to control Ta in the early morning [2]. Interestingly, this current HTa (ΔTa = 5–8°C) was sufficient to induce respiratory hypocapnia, but it was not accompanied by a change in blood pH and HCO_3_. These observations suggest that daytime HTa was sufficient to interfere with gas exchange in the lung; however, the compensation mechanism at this stage was uncovered in this study. In fact, blood pH is maintained by electroneutrality [16]. Acid is a cation, and acid loss is compensated by an increased number of other blood cations [16]. In short-term HTa, increased plasma sodium and potassium levels have been observed in pigs and chickens [17, 18]. Interestingly, supplementation with these cations decreased the percentage change in Tr during daytime HTa [7]. This supports the positive effect of these cations during daytime HTa. Therefore, the shift of the electrolyte might be responsible for the compensation during daytime HTa. Moreover, the movement of water is involved in the electrolyte; therefore, this shift might also be responsible for the different degrees of heat dissipation.

Lactating dairy goats fed under tropical requires a large amount of energy to serve heat dissipation and lactation [19]. Blood glucose is the main source of energy for both milk synthesis and respiratory muscles. There was a negative correlation between blood glucose levels and monthly THI in lactating goats fed natural HTa [20]. In addition, reactive oxygen species was a part of energy production. Both high reactive oxygen species production and depleted antioxidants result in oxidative stress. The intracellular lipid compound was susceptible to oxidative stress and was converted to MDA. Both depleted GPx and increased MDA levels were previously reported in dairy cows fed HTa [21]. Plasma GPx is mainly produced by kidney cells, especially the tubular epithelium [22]. Changes in kidney function, especially in the tubular epithelium, were also observed at the hypocapnia stage [15]. In general, the reaction of plasma GPx occurs around the cell, and the change in GPx activity can be explained by the change in production and utilization [22]. Moreover, the presence of glucose also promotes this response in cell culture [23]. In the presence of hypocapnia, the negative correlation between ΔTa and ΔGPx in the current study was accompanied by increased blood glucose levels and unchanged plasma MDA levels. This indicates the involvement of antioxidant defense in the HTa response and its relation to acid-base homeostasis. However, this response was not in line with a previous report on the long-term effects of HTa (winter vs. summer) on antioxidant defense [2]. Indeed, the difference between short-term and long-term defense has been observed in many circumstances [22].

## Conclusion

The current study revealed the effect of HTa on blood gas parameters during the summer season. In this study, ΔTa activated evaporative heat dissipation by panting and induced respiratory hypocapnia without changes in blood pH and HCO_3_. Moreover, these responses did not involve the HPA axis but were accompanied by a change in antioxidant defense.

## Authors’ Contributions

ST, DKDN, SS and NC: Conception and designed the study. DKDN, SS, and ST: Performed animal experiments. ST: Analyzed the data and performed statistical analyses. SS, ST, and SP: Wrote and revised the manuscript. ST, SS, DKDN, NC: Read and approved the final manuscript.
